# 10-Year Results of Standard Cross-Linking in Patients with Progressive Keratoconus in Romania

**DOI:** 10.1155/2019/8285649

**Published:** 2019-03-07

**Authors:** Cristina Nicula, Radu Pop, Anca Rednik, Dorin Nicula

**Affiliations:** ^1^University of Medicine and Pharmacy “Iuliu Hatieganu”, Cluj-Napoca, Romania; ^2^Oculens Clinic, Cluj-Napoca, Romania; ^3^Eye Clinic, Emergency Clinical County Hospital, Cluj-Napoca, Romania

## Abstract

**Purpose:**

The purpose of the current study was to evaluate the long-term functional results based on keratometric measurements, spherical and cylinder equivalent in patients with progressive keratoconus treated with conventional “epi-off” corneal collagen cross-linking (CXL).

**Methods:**

We conducted a retrospective study in which 113 eyes from 90 keratoconus-treated patients with CXL between 2006 and 2008 in Oculens Eye Clinic from Cluj-Napoca, Romania, were included. The diagnosis of keratoconus was based on corneal topography and its clinical signs. All patients were evaluated preoperatively, and a follow-up was performed at 1, 3, and 6 months and every year from 1 to 10 years after conventional CXL.

**Results:**

All keratometry measurements improved significantly during the follow-up. Compared to preoperative values, the improvement of *K*_max_ become statistically significant at 1 year after CXL (mean change compared to baseline −0.9 D, *p* < 0.001) and remained statistically significant thereafter up to 10 years (mean change compared to baseline −2.3 D, *p* < 0.001). As compared to preoperative values, mean spherical equivalent and mean cylinder improved during the follow-up, from a mean of −6.22 D before CXL to a mean value of −5.0 at 10 years, following CXL for spherical equivalent and from −4.4 D at baseline to −3.4 D at 10 years for cylinder (*p* < 0.05 for both). Uncorrected visual acuity increased, remaining statistically significant, by 0.104 logMAR at 10 years after CXL (*p*=0.0015), and best-corrected visual acuity increased by 0.135 logMAR at 10 years after CXL (*p*=0.015). We did not observe any case of severe complication.

**Conclusion:**

Our results show that CXL has a favorable effect on the progression of KC. The reduced *K* values, cylinder and spherical equivalent, and increased visual acuity remained the same 10 years after the procedure.

## 1. Introduction

Keratoconus (KC) is a relative frequent corneal ectasia, with prevalence in general population of 265 cases per 100,000 persons [1]. The disease is characterized by progressive thinning and ectasia of the cornea which induces irregular astigmatism, resulting in impaired vision [2]. The management of KC has mainly consisted of visual rehabilitation using glasses, contact lenses, and intracorneal ring segments implantation for moderate stages and lamellar or penetrating keratoplasty for advanced ones [3]. In 2003, corneal collagen cross-linking (CXL) was introduced by Wollensak et al. from Germany as the first therapy targeting the pathogenetic mechanisms of KC and aiming at slowing down or halting its progression [4].

CXL procedure implies photopolymerization of the stromal fibrillar tissue by combined action of a photosensitizing substance (riboflavin or vitamin B2) and irradiation with ultraviolet (UVA) light at 365 nm performed with an illuminator [4]. The purpose is the increase in stiffness and resistance of stromal fibrillar tissue to corneal ectasia and the correction of the pathogenetic causes of KC by changing intrinsic biomechanical properties of the corneal collagen [5]. The standard epithelium-off CXL protocol using a 3 mW/cm^2^ irradiance for 30 minutes was the first used for progressive KC therapy [4]. Research studies showed that this procedure increases the biomechanical strength of the cornea by photochemical cross-linking of individual collagen fibers [5].

In short term, CXL produces morphological changes which consist of an early apoptosis of keratocytes to a depth of 300 microns, stromal edema, loss of subepithelial nerve plexus and midstromal nerve fibers, and increased reflectivity of midstroma [6–8]. At functional level these morphological changes are correlated with an initial reduced visual acuity and worsening of mean *K* values [7]. Starting from month 3 after CXL, appears a keratocytic repopulation associated with regeneration of the nerve plexus and formation of lamellar compaction, epithelial thickening, and compaction of new collagen corresponding to an improvement of visual acuity [7]. Lacunar edema around apoptotic keratocytes, keratocytic repopulation, and an increased reflectivity of the extracellular matrix are the basis for the postoperative anterior stromal haze, and a demarcation line is noticed in most patients 1–5 months postoperatively [9, 10]. An increase of corneal rigidity, higher in the anterior stroma as compared to posterior stroma, was also reported after CXL, leading to an increased biomechanical stability of the cornea [11]. The development of a measurement of the biomechanical properties of the cornea in a standardized fashion could improve the surgical planning [12].

The purpose of the current study was to evaluate long-term functional results based on keratometric measurements, spherical and cylinder equivalent, in patients with progressive KC at 10 years following the therapy with conventional “epi-off” CXL technique.

## 2. Materials and Methods

We conducted a retrospective study at Oculens Eye Clinic, Cluj-Napoca, Romania. 113 eyes of 90 patients with progressive KC stages 1, 2, 3, and 3/4 (according to Amsler–Krumeich classification; stage 1: 6.2%; stage 1/2: 20.4%; stage 2: 38.1%; stage 2/3: 23.9%; stage 3: 7.1%; stage 3/4: 4.4%) treated from 2006 to 2008 by conventional “epi-off” CXL were included in this evaluation. The protocol was previously described in our 7-years report [13].

Additional inclusion criteria were a follows: age between 15 and 54 years, any gender, and average corneal thickness of at least 400 *μ*m. The diagnosis of KC was based on corneal topography, tomography, and its clinical signs such as stromal thinning, Fleischer ring, or Vogt's striae. The inclusion criteria for progression included ≥2 lines of best-corrected visual acuity (BCVA) attributable just to KC progression in addition to at least one of the following over the preceding 12 months: an increase of at least 1 diopter (D) in the steepest keratometry value derived from computerized topography or an increase in astigmatism determined by manifest subjective refraction of at least 1 D.

Patients with corneal thickness under 400 *μ*m, endothelial cell density less than 2000 cells/mm^2^, previous intracorneal segments, axial corneal scarring, Vogt's striae, herpetic keratitis or other ocular infections, dry eye syndrome, aphakia, autoimmune disease, or pregnancy were excluded.

Patients were followed in clinic on day 1, day 3, month 1, month 6, year 1, and yearly thereafter up to 10 years. At each visit, uncorrected visual acuity (UCVA), BCVA, refractometry, keratometry, corneal tomography, corneal topography, and ultrasonic pachymetry were performed.

The study was conducted in accordance with the Declaration of Helsinki (1964). An informed consent was obtained from all patients undergoing the CXL procedure. The study was approved by the clinic's ethics committee. In Romania, this procedure is not covered by the National Health Insurance; therefore, patients agreed to cover the costs of the procedure on their own expenses.

### 2.1. Procedures Performed

Before CXL procedure, all patients underwent a full ocular assessment, including the preoperative UCVA and BCVA measured as logarithm of minimum angle of resolution (logMAR scale), refractometry, keratometry (*K*_max_, *K*_min_, and *K*_avg_) using the autorefractometer and the Oculus Pentacam topographer, slit-lamp examination (Slit-Lamp BX 900, Haag-Streit AG), intraocular pressure, corneal pachymetry (performed with the Oculus Pentacam topographer), corneal topography and tomography using the Oculus Pentacam topographer (Oculus Pentacam Scheimpflug imager), and specular microscopy (Topcon SP 3000P) for counting the endothelial corneal cells. Patients were requested to discontinue contact lens wear 2 weeks before evaluation. As the anterior segment ocular coherence topographer (AS-OCT) was not available onsite at the beginning of the study, the depth of demarcation line was not assessed in all patients, and thus we did not include this parameter in the analysis.

CXL procedure was performed in the operating room in sterile conditions. The eye was prepared with topical anesthesia—alkaline solution 3-4 drops 15–20 minutes before CXL. Corneal debridement on a 9 mm diameter optical zone was performed, and corneal pachymetry was performed after epithelium removal to ascertain that it was more than 400 microns. M-standard riboflavin 0.1% (without dextran solution) was instilled every 3 minutes for 30 minutes before irradiation was performed. The central part of the cornea was further exposed at the UV light (365 nm; Pescke CXL System, System Vision), and instillation of riboflavin every 3 minutes was continued for 30 minutes, under a power of 3 mW/cm^2^. At the end of the procedure, a bandage contact lens was applied. The patient was instructed to use antibiotics and steroids as Tobradex (Alcon Novartis, Dallas Forth Worth, USA) 5 times/day for one month. The therapeutic contact lens was removed after corneal healing (3-4 days)

### 2.2. Statistical Analysis

Data were presented as mean ± standard deviation or number (frequency). Results at different time points were compared using the paired *t*-test. A *p* value <0.05 was considered statistically significant.

## 3. Results

We included in this analysis 113 eyes from 90 patients with progressive KC. 60.2% (68 patients) of the group were men. The mean age of the patients was 26.6 ± 8.0 years, ranging between 15 and 54 years (27.4% were between 15 and 20 years old; 47.8% were between 21 and 30 years old; 21.2% were between 31 to 40 years old, and 3.5% over 41 years old).

### 3.1. Keratometry

**Figure 1:**
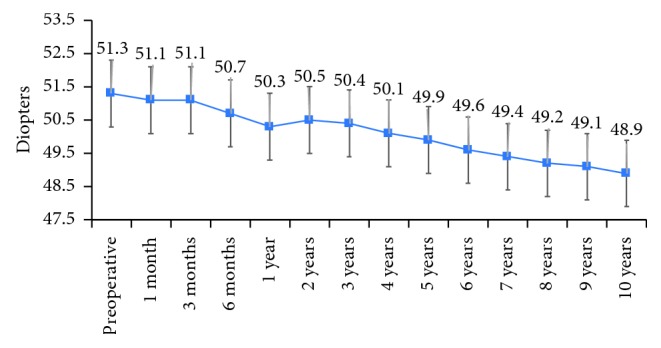
Decrease of *K*_max_ during the follow-up.

All keratometry measurements improved significantly during the follow-up. *K*_max_ decreased from 51.3 D preoperatively to 50.3 D at 1 year after CXL, stabilized between years 1 and 3, and continued to decrease thereafter up to 48.9 at 10 years (*p* < 0.001 for all time points from year 4 to year 10 compared to *K*_max_ at year 3; Figure 1). Compared to preoperative values, the improvement of *K*_max_ become statistically significant at 1 year after CXL (mean change compared to baseline −0.9 D) and remained statistically significant thereafter up to 10 years. The improvement of *K*_min_ become significant at 6 months and remained significant thereafter up to the end of the follow-up. For *K*_avg_, the improvement was statistically significant at 1 month after CXL (mean change as compared to baseline −0.2 D, *p*=0.038) and lost its significance at 3 months (*p*=0.070); as of month 6, the change became statistically significant (mean change compared to baseline −0.6, *p*=0.002) and remained significant until the end of the follow-up period (mean change compared to baseline −2.3, *p* < 0.001) (Table 1).

### 3.2. Spherical Equivalent

**Figure 2:**
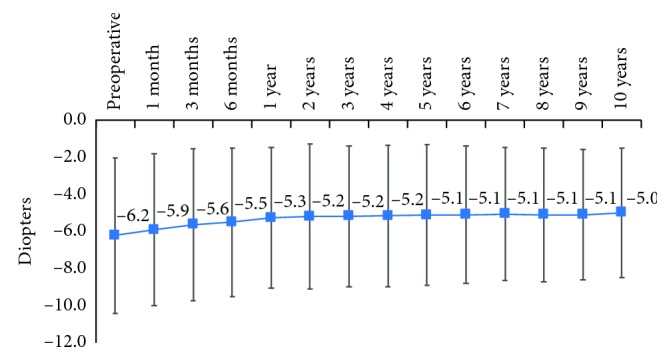
Evolution of spherical equivalent during the 10-year follow-up.

Mean spherical equivalent improved during the follow-up, from a mean value of −6.22 D before CXL to a mean value of −5.0 ± 3.5 D at 10 years following CXL. As compared to baseline, the improvement was statistically significant as of 6 months following the procedure and remained statistically significant thereafter until the end of follow-up (*p* value <0.05; Figure 2).

### 3.3. Cylinder

**Figure 3:**
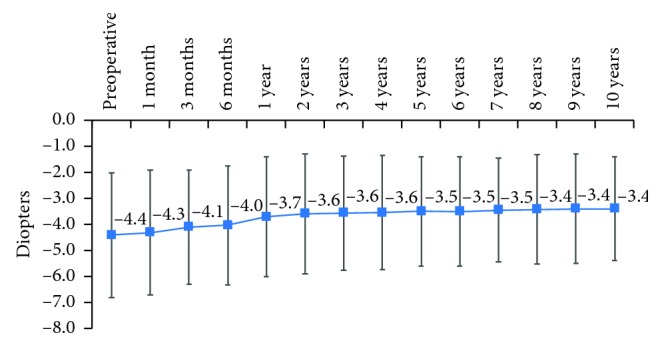
Cylinder evolution during the 10-year follow-up.

As compared to preoperative values, mean cylinder decreased from −4.4 ± 2.4 D at baseline to −3.4 ± 2.0 D at 10 years (*p*=0.015). The improvement was rapid in the first year following the CXL and stabilized thereafter until the end of follow-up (Figure 3).

### 3.4. Visual Acuity

UCVA increased statistically significant by 0.104 logMAR, from an average of 0.388 before CXL to 0.492 at 10 years after CXL (*p*=0.0015). BCVA increased by 0.135 logMAR from an average of 0.629 before CXL to 0.764 at 10 years after CXL (*p*=0.015).

### 3.5. Safety

A temporary haze appeared in 90% of cases but disappeared after 3 to 6 months. We had 6 cases of sterile infiltrates, with a positive evolution after steroids instillation. One case of delayed corneal healing was observed, with a good evolution and BCVA of 0.1 logMAR after 1 year following the CXL. There was no case of microbial, viral, fungal keratitis, or corneal melting.

## 4. Discussion

In this long-term 10-year follow-up study, we showed that conventional CXL effectively stabilized the progression of KC, as assessed by key corneal topographic parameters. In our study, *K*_max_ had a rapid decrease from 1 month to 1 year, stabilized between year 1 and year 3 and continued to decrease thereafter up to the end of the 10-year follow-up. The decrease in *K*_max_ become significant at 1 year after the CXL and remained statistically significant as compared to baseline thereafter up to the end of the follow-up. Furthermore, in our sample, we did not observe any regression with stable lower *K*_max_ values as compared to preoperative values.

Our results confirm previous long-term studies, including our previous report at 7 years [2, 13–15]. In the literature, we were able to identify only 2 studies reporting the 10-year follow-up after CXL in patients with KC. In adults, Romero-Jiménez et al. [2] also reported long-term stabilization of KC with improvement in keratometry which was maintained at 10 years. In this study, 34 eyes were enrolled and *K*_max_ decreased from 53.2 D preoperatively to 49.56 D at 10 years (*p* < 0.001) and *K*_min_ from 47.5 D to 45.5 D (*p* < 0.001). In the pediatric population, although CXL slows the progression of KC, as of 9 years following the CXL, a regression of keratometry results was shown [14]. Mazzotta et al. [14] reported the CXL results in 62 eyes of 47 pediatric patients with KC with a mean age at baseline of 14.1 years. In this pediatric group, *K*_max_ was statistically significantly lower starting from 6 months after conventional CXL (*K*_max_ = −1.07, *p*=0.0454) and maintained statistically significantly lower until the 8th year of follow-up (delta *K*_max_ = −1.07, *p*=0.0454. At year 9, delta *K*_max_ increased to −0.8 (*p*=0.1971 for the difference vs baseline) and to −0.88 at year 10 (*p*=0.445).

Our results on spherical equivalent and cylinder values showed an improvement until 1^st^ year after CXL which then stabilized and remained at 10 years statistically significantly lower as compared to baseline. These results confirm previous observations that showed an improvement in these parameters following CXL. Khattak et al. [16] in their study regarding the spherical equivalent of treatment group reported a hyperopic shift by 0.49 D (*p*=0.794) in the immediate postoperative period at 1 month. By 12 months, hyperopic shift was 0.024 (*p*=0.935), whereas control group had a myopic shift by 0.29 D (*p*=0.398) over one year. Hashemi et al. [17] in a study on long-term results in CXL procedure showed that mean spherical equivalent changed from −3.18 + −0.28 D at baseline to −2.77 + −2.18 D at 5 years following CXL, and mean refractive cylinder error changed from 3.14 ± 2.22 D at baseline to 2.49 ± 1.71 D (*p*=0.089) at 5 years following the CXL.

We also observed a significant functional improvement at 10 years with improvement of both UCVA and BCVA as compared to baseline (*p* < 0.05 at 10 years as compared to baseline). These results confirm our previous report in 114 cases in which we showed that at 7 years following the CXL, the UCVA increased from 0.78 to 0.68 logMAR [13]. A statistically significant improvement at 10 years was also reported by Mazzotta et al. [14] in 80% of the children evaluated. Similar positive results were reported by Romero-Jiménez et al. [2] who showed an improvement of corrected distance visual acuity with 0.14 logMAR (*p*=0.002) at 10 years following conventional CXL in adults with KC. These long-term results suggest that functional improvement observed in the first years after the CXL is maintained for long term in both adults and children.

In the last 6 years, the accelerated “epi-off” cross-linking technique has been used more widely as an alternative to standard CXL with the aim of decreasing the time of the procedure and thus decreases the patients' discomfort [18]. Clinical investigations showed that accelerated cross-linking is well tolerated, with no statistically significant difference in corneal stromal demarcation line depth and efficacy on short- and midterm as compared to the standard protocol [19, 20]. However, its clinical efficacy still needs to be determined in long-term follow-up and large cohort of patients.

In terms of safety, we had no case of permanent haze in our sample. Transient haze was reported in 90% of the cases during the first 6 months. We also had 6 cases of sterile infiltrates, with a positive evolution after steroids instillation and 1 case of delayed corneal healing. However, we did not observe any case of severe complication such as corneal melting. The presence of transient haze has been previously reported for the first 12 months after the CXL, but they do not have a significant impact on the final visual acuity [14]. Also, the wound-healing issues and noninfectious keratitis are among adverse events that have been previously reported after CXL procedure but without long-term consequences on visual acuity [2].

## 5. Conclusions

Our results show that CXL slows or stops the progression of KC and could reduce the need for corneal transplantation in these patients. The results were stable, consisting in reduction of *K* values, cylinder and spherical equivalent, and increased visual acuity which maintained at 10 years after the procedure.

## Figures and Tables

**Table 1:** Evolution of keratometry during the follow-up.

	*K*_min_	*p*	*K*_max_	*p*	*K*_avg_	*p*
Preoperative	47.2 ± 4.1		51.3 ± 4.8		49.3 ± 4.1	
Δ1 month	−0.2	0.159	−0.2	0.285	−0.2	0.038
Δ3 months	−0.3	0.098	−0.2	0.411	−0.2	0.070
Δ6 months	−0.8	<0.001	−0.5	0.053	−0.6	0.002
Δ1 year	−0.9	<0.001	−0.9	<0.001	−0.9	<0.001
Δ2 years	−0.8	<0.001	−0.8	0.001	−0.8	<0.001
Δ3 years	−0.9	<0.001	−0.9	<0.001	−0.9	<0.001
Δ4 years	−1.2	<0.001	−1.2	<0.001	−1.2	<0.001
Δ5 years	−1.3	<0.001	−1.4	<0.001	−1.4	<0.001
Δ6 years	−1.6	<0.001	−1.7	<0.001	−1.7	<0.001
Δ7 years	−1.8	<0.001	−1.9	<0.001	−1.8	<0.001
Δ8 years	−2.0	<0.001	−2.0	<0.001	−2.0	<0.001
Δ9 years	−2.1	<0.001	−2.2	<0.001	−2.2	<0.001
Δ10 years	−2.3	<0.001	−2.3	<0.001	−2.3	<0.001

## Data Availability

The data used to support the findings of this study have not been made available due to EU General Data Protection Regulation 2016/679. The informed consent process did not include the share of individual raw data.

## References

[1] Godefrooij D. A., de Wit G. A., Uiterwaal C. S., Imhof S. M., Wisse R. P. L. (2017). Age-specific incidence and prevalence of keratoconus: a nationwide registration study. *American Journal of Ophthalmology*.

[2] Romero-Jiménez M., Santodomingo-Rubido J., Wolffsohn J. S. (2010). Keratoconus: a review. *Contact Lens and Anterior Eye*.

[3] Raiskup F., Theuring A., Pillunat L. E., Spoerl E. (2015). Corneal collagen crosslinking with riboflavin and ultraviolet-A light in progressive keratoconus: ten-year results. *Journal of Cataract & Refractive Surgery*.

[4] Wollensak G., Spoerl E., Seiler T. (2003). Riboflavin/ultraviolet-A-induced collagen crosslinking for the treatment of keratoconus. *American Journal of Ophthalmology*.

[5] Wollensak G., Spoerl E., Seiler T. (2003). Stress-strain measurements of human and porcine corneas after riboflavin-ultraviolet-A-induced cross-linking. *Journal of Cataract & Refractive Surgery*.

[6] Wollensak G., Spoerl E., Wilsch M., Seiler T. (2004). Keratocyte apoptosis after corneal collagen cross-linking using riboflavin/UVA treatment. *Cornea*.

[7] Mazzotta C., Caporossi T., Denaro R. (2012). Morphological and functional correlations in riboflavin UV A corneal collagen cross-linking for keratoconus. *Acta Ophthalmologica*.

[8] Knappe S., Stachs O., Zhivov A., Hovakimyan M., Guthoff R. (2011). Results of confocal microscopy examinations after collagen cross-linking with riboflavin and UVA light in patients with progressive keratoconus. *Ophthalmologica*.

[9] Mazzotta C., Balestrazzi A., Baiocchi S., Traversi C., Caporossi A. (2007). Stromal haze after combined riboflavin-UVA corneal collagen cross-linking in keratoconus: in vivo confocal microscopic evaluation. *Clinical & Experimental Ophthalmology*.

[10] Wollensak G., Herbst H. (2010). Significance of the lacunar hydration pattern after corneal cross-linking. *Cornea*.

[11] Kohlhaas M., Spoerl E., Schilde T., Unger G., Wittig C., Pillunat L. E. (2006). Biomechanical evidence of the distribution of cross-links in corneastreated with riboflavin and ultraviolet A light. *Journal of Cataract & Refractive Surgery*.

[12] Stojanovic A., Zhang J., Chen X., Nitter T. A., Chen S., Wang Q. (2010). Topography-guided transepithelial surface ablation followed by corneal collagen cross-linking performed in a single combined procedure for the treatment of keratoconus and pellucid marginal degeneration. *Journal of Refractive Surgery*.

[13] Nicula C., Nicula D., Pop R. N. (2017). Results at 7 years after cross-linking procedure in keratoconic patients. *Journal Français d’Ophtalmologie*.

[14] Mazzotta C., Traversi C., Baiocchi S. (2018). Corneal collagen cross-linking with riboflavin and ultraviolet A light for pediatric keratoconus. *Cornea*.

[15] Poli M., Lefevre A., Auxenfans C., Burillon C. (2015). Corneal collagen cross-linking for the treatment of progressive corneal ectasia: 6-year prospective outcome in a French population. *American Journal of Ophthalmology*.

[16] Khattak A., Nakhli F. R., Cheema H. R. (2015). Corneal collagen crosslinking for progressive keratoconus in Saudi Arabia: one-year controlled clinical trial analysis. *Saudi Journal of Ophthalmology*.

[17] Hashemi H., Seyedian M. A., Miraftab M., Fotouhi A., Asgari S. (2013). Corneal collagen cross-linking with riboflavin and ultraviolet A irradiation for keratoconus. *Ophthalmology*.

[18] Chow V. W. S., Chan T. C. Y., Yu M., Wong V. W. Y., Jhanji V. (2015). One-year outcomes of conventional and accelerated collagen cross-linking in progressive keratoconus. *Scientific Reports*.

[19] Mazzotta C., Paradiso A. L., Baiocchi S., Caragiuli S., Caporossi A. (2013). Qualitative investigation of corneal changes after accelerated corneal collagen cross-linking (A-CXL) by in vivo confocal microscopy and corneal OCT. *Journal of Clinical & Experimental Ophthalmology*.

[20] Spadea L., Tonti E., Vingolo E. (2016). Corneal stromal demarcation line after collagen cross-linking in corneal ectatic diseases: a review of the literature. *Clinical Ophthalmology*.

